# Parkinson’s disease screening using a fusion of gait point cloud and silhouette features

**DOI:** 10.1371/journal.pone.0315453

**Published:** 2025-01-03

**Authors:** Tee Connie, Timilehin B. Aderinola, Jia You Ong, Thian Song Ong, Michael Kah Ong Goh, Bayu Erfianto, Bedy Purnama, Ming De Lim, Nor Izzati Saedon

**Affiliations:** 1 Faculty of Information Science and Technology, Multimedia University, Malacca, Malaysia; 2 School of Computer Science, University College Dublin, Dublin 4, Ireland; 3 School of Computing, Telkom University, Kabupaten Bandung, Jawa Barat, Indonesia; 4 Department of Medicine, Faculty of Medicine, Universiti Malaya, Kuala Lumpur, Malaysia; BMS Institute of Technology and Management, INDIA

## Abstract

Parkinson’s Disease (PD) is a neurodegenerative disorder that is often accompanied by slowness of movement (bradykinesia) or gradual reduction in the frequency and amplitude of repetitive movement (hypokinesia). There is currently no cure for PD, but early detection and treatment can slow down its progression and lead to better treatment outcomes. Vision-based approaches have been proposed for the early detection of PD using gait. Gait can be captured using appearance-based or model-based approaches. Although appearance-based gait contains comprehensive features, it is easily affected by factors such as dressing. On the other hand, model-based gait is robust against changes in dressing and external contours, but it is often too sparse to contain sufficient information. Therefore, we propose a fusion of appearance-based and model-based gait features for PD prediction. First, we extracted keypoint coordinates from gait captured in videos and modeled these keypoints as a point cloud. The silhouette images are also segmented from the videos to obtain an overall appearance representation of the subject. We then perform a binary classification of gait as normal or Parkinsonian using a novel fusion of the gait point cloud and silhouette features, obtaining AUC up to 0.87 and F1-Scores up to 0.82 (precision: 0.85, recall: 0.80).

## Introduction

Parkinson’s Disease is a neurodegenerative disorder with several causes and clinical symptoms. Most commonly, it is accompanied by slowness of movement (bradykinesia) [1] or gradual reduction in the frequency and amplitude of repetitive movements (hypokinesia). PD can also progress to stages where any movement is difficult (akinesia). PD is also characterized by tremors, rigidity, and general posture and gait impairment. PD patients have a reduced ability to perform activities of daily living, and hence, have a lower quality of life [2]. Although there is currently no cure for this progressive disease, early detection and interventions can slow down the progression of the disease and lead to better treatment outcomes. However, one of the main challenges with early detection of PD is the long waiting time for specialist consultation. To solve the problem of waiting times, Artificial Intelligence (AI) systems have been proposed [3] such that outpatients can undergo pre-screening before visiting a specialist. Such systems could potentially reduce the burden on healthcare systems and help slow down the progression of degenerative diseases such as PD.

In recent years, AI has been increasingly used in vision-based approaches to diagnose disease from medical images, such as x-rays [4], CT scans [5], and MRIs [6]. The main advantage of using AI for disease diagnosis is its ability to analyze large amounts of medical data quickly and accurately, providing doctors with more precise and efficient diagnostic tools. It can be used to detect and classify abnormal patterns in medical images that may indicate the presence of a disease. AI-based approaches to disease diagnosis are still in their early stages and there are many challenges to be addressed, such as the need for large amounts of high-quality annotated medical data, as well as ensuring the accuracy and interpretability of AI models [7]. However, the potential benefits of using AI in disease diagnosis are significant, including earlier detection, more accurate diagnoses, and more personalized treatment plans. For example, AI techniques were proposed for the pre-screening of COVID-19 from cough recordings [8, 9], and a technique was proposed for early PD detection using pre-motor features [10].

In general, using AI for pre-screening involves data capture, feature extraction based on early stage symptoms, and classification. Important attributes of a pre-screening system include accessibility and ease of use, where patients do not require expert assistance for data capture, for example, using cough recordings for COVID-19 pre-screening. Another important factor is whether the disease presents observable symptoms at the early stages. For example early signs of PD can be observed in gait, including reduced arm swing amplitude and symmetry, gait speed, step length, and increased time spent in the double-support phase [11]. The visible motor signs of Parkinson’s disease are referred to as Parkinsonism [12].

Recently, vision-based approaches have been proposed to detect potential Parkinsonism in gait [13]. This has been enabled by pose estimation models such as AlphaPose [14], which automatically tracks subjects of interest in videos and outputs coordinates of anatomical points on their bodies. Although Parkinsonism exists in some other gait pathology [2], a vision-based system could serve as a gait pre-screening system to reduce clinical consultation costs and waiting times. There are two common approaches to vision-based gait capture, namely, model-free (also referred to as appearance-based) and model-based [15–17]. The model-free approach relies on binary images segmented from images. Although model-free gait contains comprehensive features, it is easily affected by factors such as view angles, clothing changes and carrying changes. On the other hand, the model-based approaches measure gait parameters using skeleton models of the body. Model-based gait is robust against changes in dressing and eliminates the influence of external contours [18]. However, model-based gait features are too sparse to contain sufficient information for recognition—the extracted keypoints are too limited to reflect the complete gait information.

Hence, we propose FuGaPS (Fusion of Gait Point Cloud and Silhouette), a vision-based approach to diagnosing PD using both model-free and model-based gait. FuGaPS retains the human body structure and is robust to dress or carrying variances. It also contains richer information than existing model-based methods using human keypoints. First, we extracted keypoint coordinates from gait captured in videos using an AlphaPose pretrained model. Then we model these keypoints as a point cloud, which provides a richer representation, robust against small perturbations and noise in the pose estimation coordinates [19]. We also use the pretrained model of DeepLabv3+ [20] to obtain silhouette images from the videos. We then perform a binary classification of gait as normal or Parkinsonism using FuGaPS, a fusion of the gait point cloud and silhouette features. To the best of our knowledge, this is the first attempt at using a fusion of gait point clouds and silhouettes for PD detection.

### Related works

PD screening usually involves data capture, feature extraction based on PD-related patterns, and classification. Brain electroencephalogram (EEG) signals [21] are commonly used for PD detection. Although EEG features are accurate, EEG can only be captured with specialized equipment and requires expert knowledge for interpretation. Since PD also affects speech, voice signals of patients have also been used for PD detection [22]. However, changes to the voice may be subtle. In addition, voice is easily affected by noise and may require advanced signal processing techniques [23]. Handwriting has also been used for PD detection [24, 25]. However, handwriting cannot be obtained without the cooperation of subjects.

Gait is perhaps the most used external feature for PD detection because it can be captured unobtrusively using force plates, vision-based techniques, or wearable sensors. For example, ground reaction forces have been used for PD detection using machine learning techniques [26]. However, force plates are relatively expensive and require expert knowledge to operate. Other techniques have also been proposed [27], which rely on body markers for gait signal acquisition. However, patients may be affected by the presence of markers on their bodies. Therefore, markerless techniques have been proposed for PD screening [13], which rely on vision-based gait capture enabled by state-of-the-art pose estimation techniques such as AlphaPose and OpenPose [28].

There are two common approaches to vision-based gait capture, namely, model-free (also referred to as appearance-based) and model-based [15–17]. For example, a model-free technique [29] was proposed for the detection of bradykinesia in PD from videos using optical flow. A model-based technique was also proposed [30] which relies on 2D and 3D poses and uses an ensemble of deep learning models to predict Unified Parkinson’s Disease Rating Scale (UPDRS) scores.

## Materials and methods

**Fig 1:**
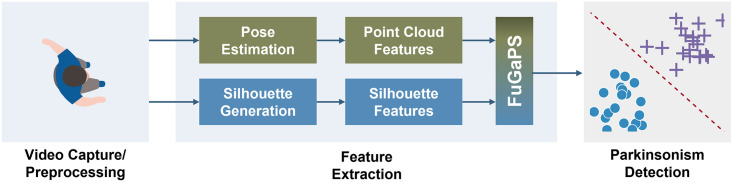
Overall methodology showing the process of FuGaPS generation and PD detection.

This section describes the techniques for gait capture, feature extraction, and Parkinsonism prediction (Fig 1).

### Dataset

The data used in this study are videos acquired from various online sources. The videos depict individuals walking with either Parkinsonian or normal gait. To ensure accuracy, the videos were appropriately annotated to indicate which ones showcase Parkinsonism. A PD expert has been invited to validate the accuracy of the annotated samples. This annotation step is crucial, as it directly influences the trained model’s precision. We collected data from 294 subjects with mean age 68±5.83 years, with 97 females. The dataset includes 150 healthy individuals and 144 individuals diagnosed with PD. Online video recordings were selected where the subject is walking and their full body is visible in the video. The dataset collection and method of analysis complied with ethical procedures and was approved by the Ethics Committee of Multimedia University with ethics approval number EA0422022.

### Feature extraction

Two different kinds of features were explored, namely, point cloud features (model-based) and silhouette features (model-free). The point cloud features were derived from the keypoint features. The keypoint features were obtained by performing pose estimation to extract the positions of the body joints of subjects from the videos. After that, the point cloud features are generated to obtain robust gait features insensitive to texture variations. On the other hand, the silhouette features were acquired by segmenting the walking subjects from the image background. The details for each approach are provided in the subsequent sections.

#### Point cloud features

**Fig 2:**
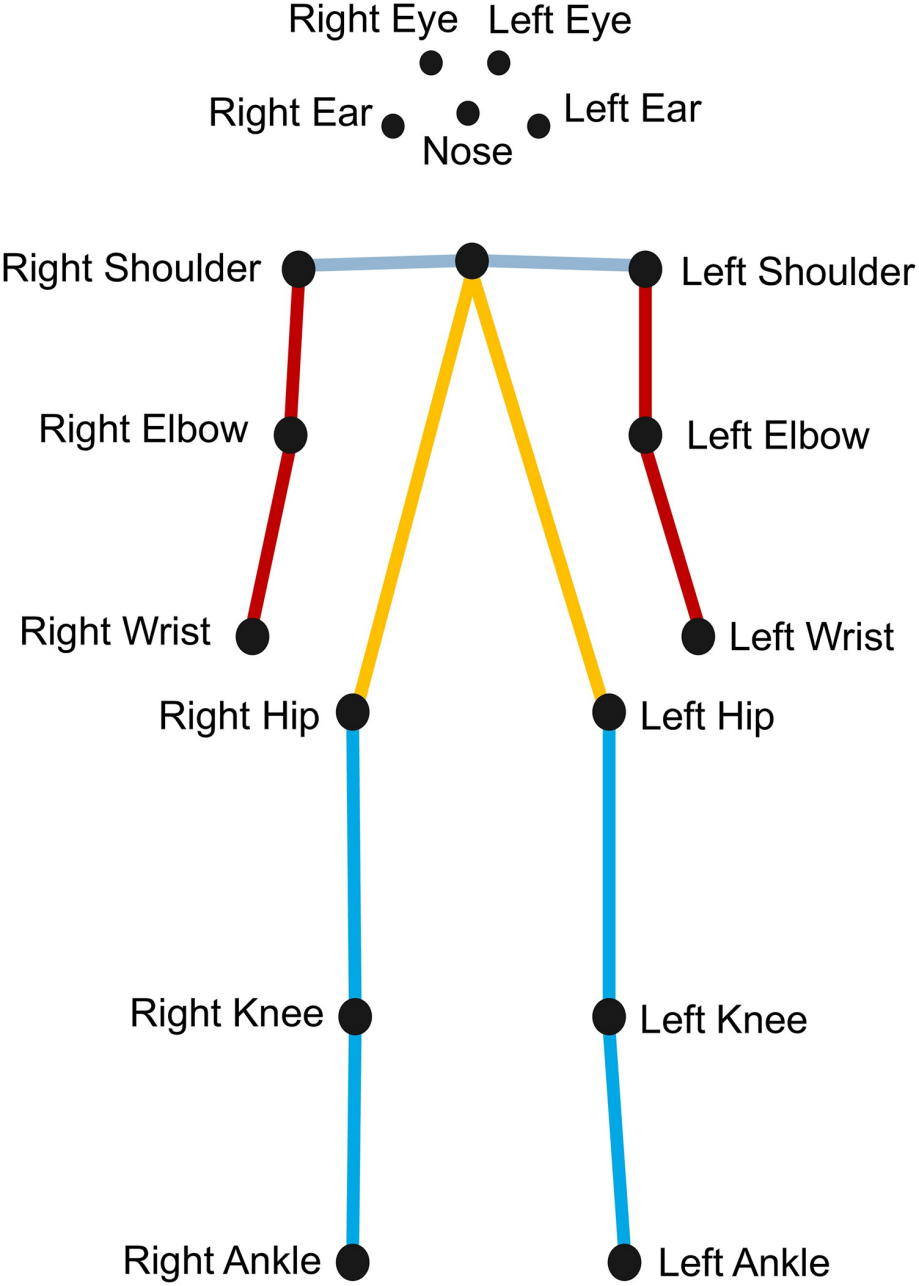
Pose estimation keypoints.

First, the AlphaPose [14] pretrained pose estimation model was used to extract body keypoints including the joint positions from each video frame. These keypoints can represent the position and movement of various body parts, such as the head, torso, and limbs (Fig 2).

After that, the extracted keypoints were preprocessed to make them suitable for model training. This involves sequencing the source video to 100 frames per sequence. For example, if the video contains 432 frames, then four sequences will be formed by taking the first 400 frames, each containing 100 frames. Each frame included the keypoints of the Parkinson’s patient which can be used for gait analysis. This processing part is required to feed video sequences with fixed lengths to the training model. For a walking sequence of *T* seconds in which we track *K* body keypoints in a video with frame rate *f*, AlphaPose outputs a sequence $\left\{\left(x_{t}^{i},y_{t}^{i},p_{t}^{i}\right)|i=1,\ldots ,K;t=1,\ldots ,fT\right\}$, where $\left(x_{t}^{i},y_{t}^{i}\right)$ is the 2D coordinates, and $p_{t}^{i}$ the confidence of the *i*^*th*^ keypoint in frame *t*. 3D keypoints are then generated by mapping the 2D keypoints to a depth map to obtain the corresponding depth coordinate for each keypoint [31].

**Fig 3:**
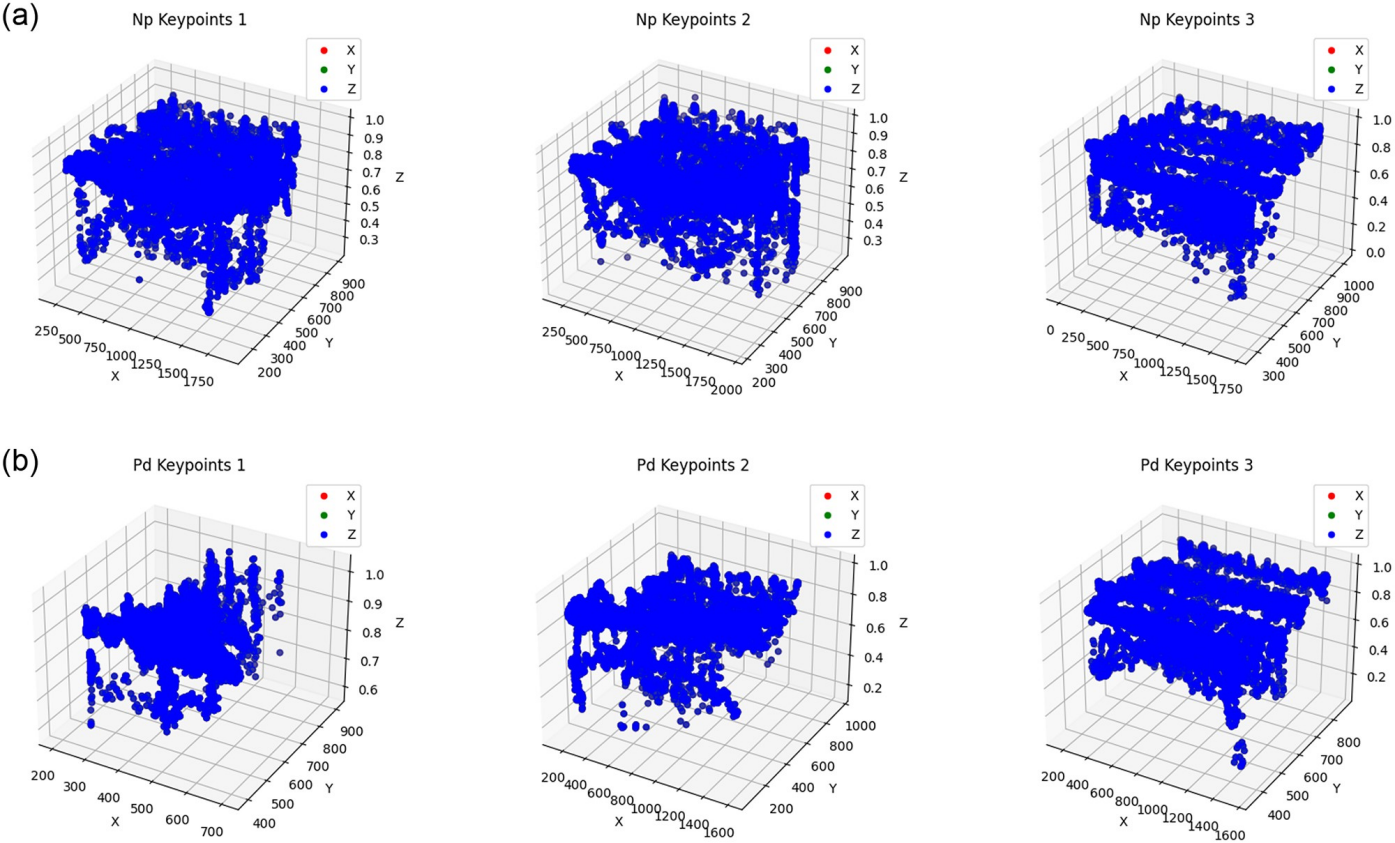
Keypoints generated using AlphaPose for (a) NP (Non-Parkinson’s Disease) (b) PD (Parkinson’s Disease) groups.

As shown in Fig 3, in the NP group, the keypoints are widely spread across a large area, indicating a wide range of motion and active movement. This extensive distribution of keypoints suggests that individuals in the NP group have normal and dynamic gait patterns without significant posture issues. Their movements are fluid, reflecting a high degree of mobility and an absence of restrictions in their bodily motions.

#### Fusion of point cloud and silhouette features

**Fig 4:**
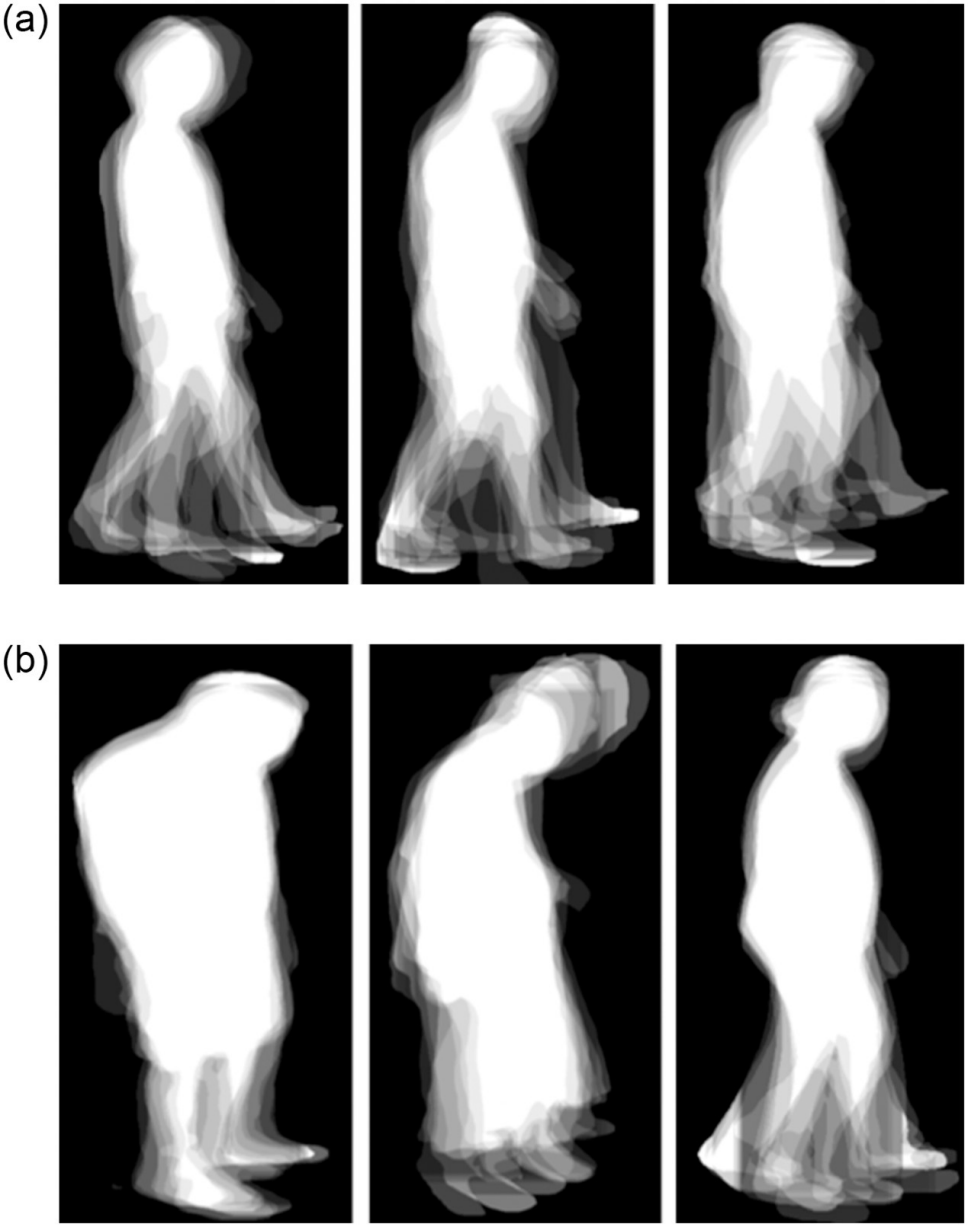
Sample gait energy images for the non-PD (NP) and PD groups. (a) NP. (b) PD.

To obtain the silhouette data, we used the pretrained model of DeepLabv3+ [20] which is a state-of-the-art semantic segmentation algorithm that can accurately segment objects and classify pixels in images. It is built upon a ResNet-50 backbone that is pretrained on the ImageNet dataset. The silhouette data is a sequence of images, with one image per video frame, which are averaged to obtain the gait energy image (GEI) for each subject (Fig 4). Each image was flattened to a vector with shape 100 × 100 × 3. Then, the fused feature was obtained by concatenating the flattened silhouette vector with the flattened point cloud features.

## Experiments and results

### Experiment setup

The experiments were performed on a machine running a 64-bit operating system with an Intel Core i5 processor, 16GB RAM, NVIDIA GeForce GTX 1650 Ti 4GB GPU. Experiments were run using Python 3.8.15. Three distinct training scenarios were implemented, applying the same parameters across all three models to evaluate their performance. In the first training scenario, AlphaPose keypoints were resized to a uniform length and organized into point clouds representing XYZ coordinates. The point clouds were flattened into 1D arrays, creating the feature set for this scenario. The second training scenario involved using only Gait Energy Images (GEI), which were resized into 1D arrays. The third and final training scenario combined the GEI images and AlphaPose keypoints into a single feature set. This was achieved by horizontally stacking the flattened GEI image arrays with the flattened AlphaPose keypoints.

**Table 1:** Results on individual and fused features.

Model	Feature	AUC	Precision	Recall	F1-score
Decision Tree	Fusion	0.75	0.82	0.64	0.72
	PointCloud	0.66	0.70	0.60	0.65
	GEI	0.59	0.66	0.42	0.51
Extra Trees	Fusion	0.71	0.83	0.30	0.44
	PointCloud	0.66	0.71	0.34	0.46
	GEI	0.57	0.52	0.22	0.31
KNN	Fusion	0.82	0.96	0.48	0.64
	PointCloud	0.69	0.65	0.66	0.65
	GEI	0.81	0.88	0.60	0.71
Logistic Regression	GEI	0.79	0.78	0.70	0.74
	PointCloud	0.60	0.68	0.52	0.59
	Fusion	**0.87**	0.85	**0.80**	**0.82**
Random Forest	PointCloud	0.69	0.77	0.40	0.53
	Fusion	0.73	0.82	0.28	0.42
	GEI	0.78	**1.00**	0.32	0.48
SVM	PointCloud	0.57	0.64	0.46	0.53
	Fusion	0.87	0.87	0.66	0.75
	GEI	0.79	0.84	0.62	0.71

Best results are shown in **bold** font face.

### Results

**Fig 5:**
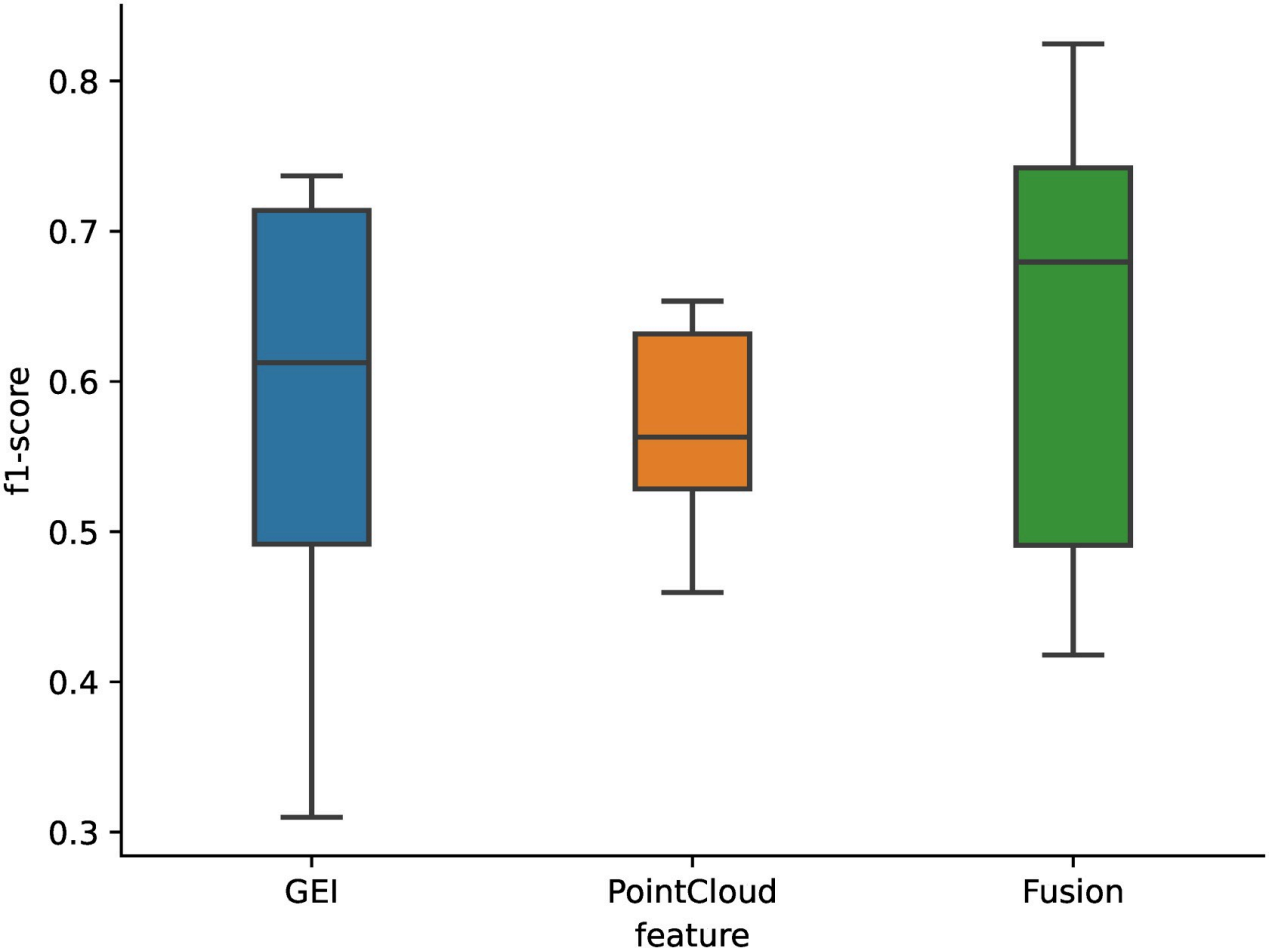
Summary of model f1-scores by feature type.

**Fig 6:**
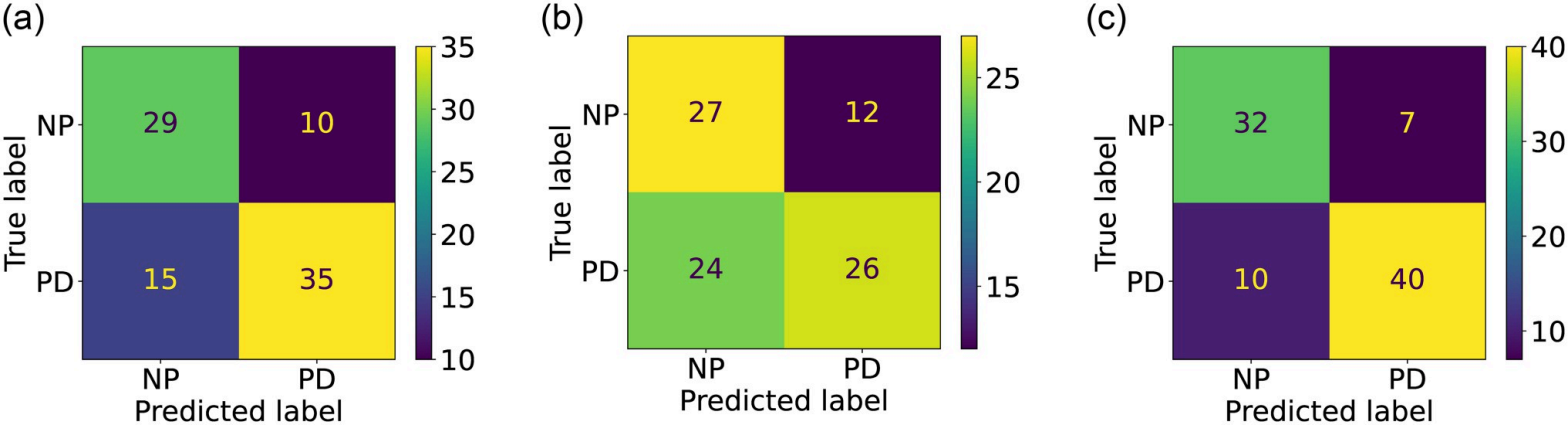
Confusion matrices for PD screening with logistic regression using (a) GEI, (b) PointCloud, and (c) GEI + PointCloud.

For each training scenario, we use a 30% test size, yielding 205 training samples (111 NP, 94 PD) and 89 test samples (39 NP, 50 PD). We evaluated our approach using Support Vector Machines (SVM), Random Forest Classifier, Extra Trees Classifier, Logistic Regression, K-Nearest Neighbors, and Decision Trees. To tune hyperparameters, we employ the Grid Search technique with five-fold cross-validation. For evaluation, we used the AUC, precision, recall, and F1-score. To get a good balance between precision and recall, we used the F1-score as our main metric As shown in Fig 5, the overall best results are obtained using the fusion of GEI and PointCloud features with Logistic Regression. The full results are shown in Table 1, and the confusion matrices for Logistic Regression are shown in Fig 6.

As shown in Table 1, the overall best results are obtained with the combined features using Logistic Regression with the highest AUC of 0.87, 80% recall, and f1-score of 0.82. The confusion matrices for Logistic Regression are shown in Fig 6.

## Conclusions and future work

In this study, we have proposed vision-based techniques for screening patients for the likelihood of Parkinson’s disease from both model-based gait and model-free gait. In the first experiment, we used gait energy images and obtained F1-scores up to 0.74 using Logistic Regression. In the second experiment, we extracted point cloud features from subjects’ joint coordinates obtained via pose estimation. This technique achieved f1-scores up to 0.65 with Decision Trees and KNN. In the third experiment, we fused the GEI silhouette feature with point cloud features and obtained F1-scores up to 0.82 using Logistic Regression. These results suggest that a fusion of model-free and model-based gait features offers great improvement over model-based gait for Parkinson’s disease screening. Using silhouettes in gait tests is very promising since most clinical gait tests are performed in indoor settings where background subtraction is not a challenge. Moreover, silhouettes will capture most posture-based features, while model-based descriptors will capture more kinematic features.

Although this study has used a modest-sized self-collected dataset, the results show the potential of using fusing model-free and model-based features for the early detection of Parkinson’s disease. This can potentially allow for early intervention and treatment, which will in turn lead to a higher quality of life for people living with Parkinson’s disease. Future research could be focused on collecting more representative data across different collaborative study sites, and also classifying more stages of PD progression, from mild, to moderate, and severe stages the disease.
